# A longitudinal neuroimaging dataset on multisensory lexical processing in school-aged children

**DOI:** 10.1038/s41597-019-0338-5

**Published:** 2019-12-20

**Authors:** Marisa N. Lytle, Chris McNorgan, James R. Booth

**Affiliations:** 1 Department of Psychology and Human Development, Vanderbilt University, Nashville, TN USA; 2 Department of Psychology, State University of New York at Buffalo, Buffalo, New York USA

**Keywords:** Functional magnetic resonance imaging, Magnetic resonance imaging, Human behaviour, Reading

## Abstract

Here we describe the open access dataset entitled “Longitudinal Brain Correlates of Multisensory Lexical Processing in Children” hosted on OpenNeuro.org. This dataset examines reading development through a longitudinal multimodal neuroimaging and behavioral approach, including diffusion-weighted and T1-weighted structural magnetic resonance imaging (MRI), task based functional MRI, and a battery of psycho-educational assessments and parental questionnaires. Neuroimaging, psycho-educational testing, and functional task behavioral data were collected from 188 typically developing children when they were approximately 10.5 years old (session T1). Seventy children returned approximately 2.5 years later (session T2), of which all completed longitudinal follow-ups of psycho-educational testing, and 49 completed neuroimaging and functional tasks. At session T1 participants completed auditory, visual, and audio-visual word and pseudo-word rhyming judgment tasks in the scanner. At session T2 participants completed visual word and pseudo-word rhyming judgement tasks in the scanner.

## Abstract (summary)

**Table:** 

Measurement(s)	reading and spelling ability • intelligence • brain • brain physiology trait
Technology Type(s)	psychoeducational test administration • magnetic resonance imaging • functional magnetic resonance imaging • Diffusion Weighted Imaging
Factor Type(s)	age • reading disability • type of task • parental educational level
Sample Characteristic - Organism	Homo sapiens

Machine-accessible metadata file describing the reported data: 10.6084/m9.figshare.11298188

## Background & Summary

Neuroimaging allows us to explore how the developing brain supports emerging skills necessary for success. Reading is one of these skills and involves the complex neural process of mapping written symbols to their spoken auditory word forms. Learning to read is critical, as having below standard literacy skill has been shown to have long-term consequences on academic, social, and economic success^1,2^. However, learning to read is difficult for some, as 5–10% of individuals are diagnosed with dyslexia^3^. Thorough longitudinal neuroimaging of reading development and the comparison of children with different levels of ability is necessary for understanding the underlying mechanisms of this important academic and life skill.

**Fig. 1:**
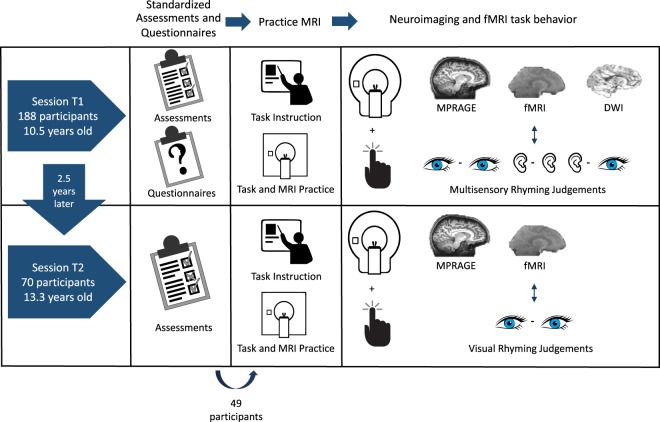
Overview of study design. Illustration of the data collected at each session, including standardized assessments and questionnaires, anatomical structural imaging (MPRAGE), functional imaging (fMRI) of rhyming judgements and diffusion weighted imaging (DWI). Note that only 49 participants were able to complete the MRI portion of the second session.

This dataset explores the brain and behavioral mechanisms of reading development through the combination of longitudinal neuroimaging and standardized psycho-educational measures of children aged 7.5- to 16.5- years old. One hundred and eighty-eight participants with a wide variety of reading and cognitive skill, including children diagnosed with reading disability by an external clinician prior to study enrollment, were recruited from the greater Chicago area for session T1. Participants were approximately 10.5 years-old at session T1 and 70 were followed up approximately two and a half years later for session T2. Prior to neuroimaging, all participants completed a battery of psycho-educational testing to quantify their reading and cognitive abilities. At session T1 parent/guardian(s) also completed questionnaires about the child’s developmental and medical history. Neuroimaging included three imaging modalities: structural MRI, diffusion-weighted imaging, and functional MRI. During functional MRI, participants completed six rhyming judgement tasks in the scanner. These rhyming judgments were either unisensory (auditory or visually presented only) or multisensory (audio-visual) and varied in lexical information, being either words or pseudo-words. Participants completed diffusion weighted imaging and tasks in the auditory and audio-visual modalities at session T1 only. Figure 1 provides an overview of the study design.

Processing of written lexical information is a multisensory process that incorporates the integration of the orthographic and phonological properties of words in order to access semantic information necessary for comprehension^4^. This dataset, by employing the same rhyming judgement task in different sensory modalities and lexicality conditions, allows for a nuanced examination of different aspects of the neural mechanisms of lexical processing. The word and pseudoword lexicality conditions tease apart the impact of semantic knowledge on the phonological rhyming judgment, as it permits identification of factors that contribute towards processing words versus pseudowords, which are pronounceable word-like letter strings that have no semantic content. The lexicality effect has been an important manipulation for testing models of word recognition^5,6^. In fact, three previous reviews have examined the patterns of word and pseudoword activations across multiple tasks^7–9^, yet none of these have examined developmental or disability differences. Additionally, the word pairs are systematically varied in orthographic and phonological similarity, resulting in pairs that have conflicting versus non-conflicting spelling and pronunciations, allowing for a parametric manipulation of difficulty.

This large longitudinal neuroimaging dataset has unique components that allow for multiple avenues of future research. The extensive phenotypic information collected in the form of standardized assessments and questionnaires allows one to explore the interplay between brain function and behavioral measures of cognitive and academic ability. For example, it is not known whether the neural basis of poor reading depends on IQ, which has been a central controversy in dyslexia^10^. These relations of brain and behavior are further supplemented by three different neuroimaging modalities, opening avenues for exploring the relation of white matter structural integrity, brain function, and behavior. Other studies have fruitfully examined fMRI and DTI relations in dyslexia^11^, and in language comprehension in children^12,13^. Finally the dataset includes 70 longitudinal subjects, 49 of which have longitudinal neuroimaging data. This longitudinal design allows for the investigation of individual change over time providing more robust measures of reading development than cross-sectional designs^14^. An additional benefit of longitudinal designs is that they allow for an examination of whether subsequent development can be predicted^15,16^.

Here we describe the public neuroimaging and behavioral dataset entitled “Longitudinal Brain Correlates of Multisensory Lexical Processing in Children” available on the OpenNeuro project (https://openneuro.org), and organized in compliance with the Brain Imaging Data Structure (BIDS). This dataset has been used, in part, in previous publications^17–28^. Our hopes in making this raw data publically accessible is to aid in openness, reproducibility, and reliability in neuroimaging research.

## Methods

### Participants

Data from 188 children were included in this longitudinal study at session T1. 108 participants were invited to return approximately two and a half years after their initial testing date. Table 1 details the time within and between sessions T1 and T2. 49 participants returned for scanning and assessments at session T2, and 21 completed assessments only due to having braces or time constraints. 28 participants were not invited back due to low performance at session T1 on the in-scanner tasks, defined as performing at less than chance on orthographically congruent trials. Subjects with low performance were not followed longitudinally due to an inability to determine if they understood and were actively performing the in-scanner tasks at session T1. Lastly, 52 participants were not invited back due to the study completing prior to their follow-up date. Table 2 provides a breakdown of reason for attrition. Sex, handedness, and age at standardized assessment session and each scan for all participants is detailed in the *participants.tsv* at the root level of the dataset. Table 3 contains a description of the number of participants at each session by task.

**Table 1:** Time between measurements. Mean, standard deviation, and range of time between measurements for within and across sessions T1 and T2.

		Time Between (years)		
		Mean	SD	Range
Within Session	Start T1–End T1	0.4	0.3	0–1.3
	Start T2–End T2	0.1	0.2	0–0.8
Across Session	Start T1–Start T2	2.7	0.6	2–4.2

**Table 2:** Number of participants per reason for study attrition. Number of participants not returning for session T2 sorted for given reason for leaving study.

Reason for Attrition	Number of Participants
Not invited back (study closed prior to T2 invitation)	52
Not invited back (poor T1 performance)	28
Not available/Busy	35
No response to invitation	7
Family moved states	3
Other	6

**Table 3:** Number of participants completing each task. Number of participants having completed one or more runs of the experimental task and sex distribution.

			Session	Number of participants		
				Female	Male	Total
fMRI	Auditory-Auditory	Word	T1	56	70	126
		NonWord	T1	54	67	121
	Auditory-Visual	Word	T1	65	73	138
		NonWord	T1	61	70	131
	Visual-Visual	Word	T1	86	92	178
			T2	21	28	49
		NonWord	T1	85	93	178
			T2	20	28	48
DWI			T1	51	62	113

Participants were recruited from the Chicago area by advertisements (public transit, magazine, google), community events, and brochures sent to schools, churches, clinics, and community organizations. Advertisements and brochures targeted children with reading difficulty or disability as well as typically developing children in an effort to recruit a diverse sample as indexed by standardized measures of reading skill. Table 4 describes the level of education of the parents. All participants were right-handed, native English speakers, with no history of psychiatric illness, neurological disease, attention deficit hyperactivity disorder (ADHD), prematurity less than 36 weeks, significant hearing loss, medication affecting central nervous system processing, or contraindications for MRI as reported by their parent/guardian. Participants and guardians were explained the details of the study and informed consent was obtained from participants and guardians including permission for de-identified data to be shared. All procedures and protocols were approved by the Institutional Review Board at Northwestern University.

**Table 4:** Parental education at session T1. Highest degree completed by mother and father as reported in developmental history questionnaire at session T1.

Highest Degree Completed	Mother	Father
No high school	7	11
High school	23	40
Some college	49	44
Bachelor’s degree	46	36
Graduate degree	40	29
Not reported	23	28

### Psycho-educational assessments and questionnaires

Participants completed a series of standardized psycho-educational assessments at both session T1 and session T2 to measure a variety of cognitive abilities. Assessments included the Comprehensive Test of Phonological Processing (CTOPP)^29^, the Test of Word Reading Efficiency (TOWRE)^30^, the Wechsler Abbreviated Scale of Intelligence (WASI)^31^, and the Woodcock-Johnson III Tests of Achievement (WJ-III)^32^. Table 5 provides a complete description of subtests administered at each session. Raw scores and age scaled or standardized scores are provided for all tests as well as composite scores when applicable. Test order was counterbalanced across participants. At session T1 only, parents/guardians completed a developmental history questionnaire and the ADHD Rating Scale IV: Home Version (adhd-rs)^33^. The developmental history questionnaire asked parents/guardians about their child’s difficulties and/or diagnosed disorders, school environment, learning preferences, parental/family demographics, and parental/family medical history. A complete list of questions on the questionnaire is included with the dataset in the accompanying data dictionary for the questionnaire, *phenotype/ses-T1/dev_hist_questionnaire.json*. Assessment and questionnaire data are located in the phenotype subdirectory and are categorized by session and then test. Data are stored as tab-separated-values tables (i.e. <test>.tsv) and are accompanied by a data dictionary describing the test and table columns (i.e. <test>.json). Table 6 includes distributions on standardized measures.

**Table 5:** Standardized psycho-educational tests and subtests completed at each session.

Measure	Test	Subtest	Session T1	Session T2	Scores
Achievement	Woodcock-Johnson III (WJ-III)	Letter-Word Identification	*	*	RS & StS
		Reading Fluency	*		
		Calculation	*		
		Spelling	*		
		Passage Comprehension	*		
		Word Attack	*	*	
		Picture Vocabulary	*		
		Oral Comprehension	*		
		Basic Reading skills	*	*	CS
Attention-deficit/hyperactivity disorder	ADHD Rating Scale-IV: Home Version	Hyperactivity and Impulsivity	*		RS
		Inattention	*		
		Total	*		
Intelligence	Wechsler Abbreviated Scale of Intelligence (WASI)	Vocabulary	*	*	RS & TS
		Block design	*	*	
		Similarities	*	*	
		Matrix reasoning	*	*	
		Verbal IQ	*	*	CS
		Performance IQ	*	*	
		Full IQ	*	*	
Phonological Processing	Comprehensive Test of Phonological Processing (CTOPP)	Elision	*	*	RS & StS
		Blending Words	*	*	
		Memory for Digits	*		
		Nonword Repetition	*		
		Rapid Digit Naming	*		
		Rapid Letter Naming	*		
		Phonemic Awareness	*	*	CS
		Phonemic Memory	*		
		Rapid Naming	*		
Reading	Test of Word Reading Efficiency (TOWRE)	Sight Word Efficiency	*	*	RS & StS
		Phonemic Decoding Efficiency	*	*	
		Total Word Reading Efficiency	*	*	CS

**Table 6:** Age at standardized testing and distribution of standardized scores for key measures. Reading Fluency corresponds to the total word reading efficiency composite score on the Test of Word Reading Efficiency (TOWRE); Phonemic Awareness corresponds to the phonemic awareness composite score on the Comprehensive Test of Phonological Processing (CTOPP); Intelligence corresponds to the full scale IQ composite score on the Wechsler Abbreviated Scale of Intelligence (WASI). The Longitudinal ST participants were those who additionally completed standardized testing at T2, and the Longitudinal MRI participants were those who additionally completed the MRI task at T2.

	Session	T1 Sample (n = 188)		Longitudinal ST Sample (n = 70)		Longitudinal MRI Sample (n = 49)	
		Mean(SD)	Range	Mean(SD)	Range	Mean(SD)	Range
Age	T1	10.5 (1.6)	7.5–14.4	10.6 (1.5)	7.5–14.4	10.5 (1.6)	7.5–14.4
	T2	—	—	13.3 (1.5)	9.5–16.3	13.2 (1.6)	9.5–16.3
Reading Fluency	T1	95.7 (17.5)	51–153	102.5 (19.5)	51–153	101.9 (17.5)	59–141
	T2	—	—	99.5 (17.3)	66–133	97.4 (16.4)	66–133
Phonemic Awareness	T1	98.2 (13.7)	67–127	103.1 (12.4)	73–127	102.8 (11.8)	73–124
	T2	—	—	100.1 (18.7)	21–127	99.9 (15.9)	37–121
Intelligence	T1	108.5 (16.1)	77–145	113.6 (16.1)	78–144	115.0 (14.8)	78–144
	T2	—	—	110.4 (15.3)	68–143	112.1 (14.8)	88–143

### Practice imaging

All participants completed a practice MRI session in a mock scanner at least once prior to the first imaging session at both time points. The practice session allowed participants to become familiar with the in-scanner tasks as well as the scanning environment. The practice session was used to reduce participant anxiety when completing the real MRI, train participants on remaining still in the scanner, and increase participant’s task understanding. In each practice session, participants were first presented with a PowerPoint explanation of all tasks and then completed practice versions of each task in the mock scanner. Each practice task consisted of 48 word pair trials including 12 from each condition, 24 fixation control trials, and 12 perceptual control trials. Detailed descriptions of trial type and timing is located in the functional task description. No word pairs used in the practice tasks were used in the functional imaging tasks.

### Imaging acquisition

All neuroimaging data were collected using a 3T Siemens Trio-Tim scanner, Siemens Syngo software version MR B17, located at Northwestern University Center for Advanced Magnetic Resonance Imaging (CAMRI). All images were acquired using a 16-channel head coil. Participants were positioned supine in the MRI scanner and foam pads were placed around the head to minimize movement. Participants were given a right hand response box to respond to functional imaging tasks. All stimuli were projected on a screen behind the scanner which participants viewed in a mirror attached to the head coil. Audio stimuli were presented through sound attenuating headphones to minimize the effects of scanner noise. During structural MRI and diffusion weighted imaging participants watched a movie to increase comfort. Participants were encouraged to remain still and were given breaks to talk to the experimenter between scans.

#### Structural MRI

T1-weighted MPRAGE images were collected using the following parameters: TR = 2300 ms, TE = 3.36 ms, matrix size = 256 × 256, bandwith = 240 Hz/Px, slice thickness = 1 mm, number of slices = 160, voxel size = 1 mm isotropic, flip angle = 9°.

#### Functional MRI

Blood oxygen level dependent signal (BOLD) was acquired using a T2-weighted susceptibility weighted single-shot echo planar imaging (EPI) and the following parameters: TR = 2000 ms, TE = 20 ms, matrix size = 128 × 120, bandwidth = 1302 Hz/Px, slice thickness = 3 mm (0.48 mm gap), number of slices = 32, voxel size = 1.7 × 1.7 × 3.0 mm, flip angle = 80°, GRAPPA acceleration factor = 2. Slices were acquired interleaved from bottom to top with even slices acquired first. 202 volumes were acquired in each run and the first 6 were removed to allow for equilibration resulting in 196 volumes per run for all tasks.

#### Diffusion weighted imaging

Diffusion weighted images were collected using echo-planar spin echo imaging and the following parameters: TR = 9400, 9500, or 9512 ms, TE = 89 ms, matrix size = 128 × 128, bandwidth = 1346 Hz/Px, slice thickness = 2 mm, number of slices = 72, voxel size = 2 mm isotropic, flip angle = 90°, GRAPPA acceleration factor = 2, 1 *b* = 0 s/mm^2^, 64 non-collinear diffusion-encoding directions *b* = 1000 s/mm^2^. TR was adjusted over the course of data acquisition. TR for each image is included in the *sub-*<*ID*>*_ses-T1_dwi.json* file alongside the diffusion weighted nifti image file in each participant’s folder.

### Functional MRI tasks

**Fig. 2:**
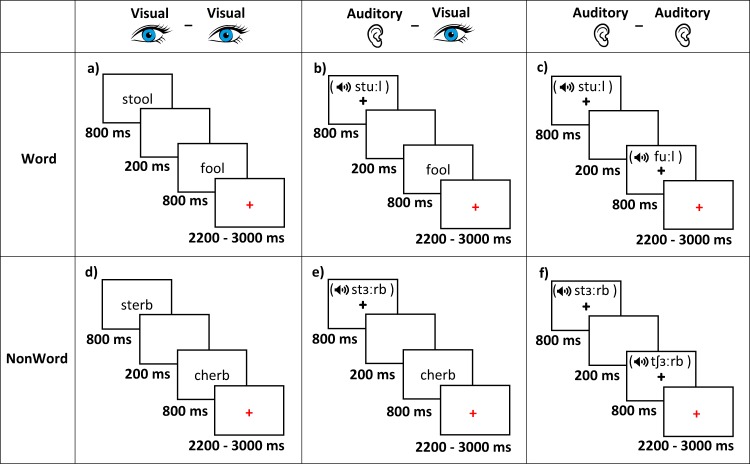
Lexical trials stimuli and timing. Illustration of the lexical stimuli and timing for (**a**) visual-visual word rhyming judgement task, (**b**) auditory-visual word rhyming judgement task, (**c**) auditory-auditory word rhyming judgement task, (**d**) visual-visual non-word rhyming judgement task, (**e**) auditory-visual non-word rhyming judgement task, and (**f**) auditory-auditory non-word rhyming judgement task.

Participants completed six in-scanner rhyming judgment tasks at session T1. Tasks varied by lexicality, containing either English words (e.g., *stool*) or pseudo-words, which are pronounceable but meaningless word-like letter strings (e.g., *sterb*), and by sensory modality. Stimulus pairs were either presented auditorily (AA), visually (VV), or with the first item presented auditorily and the second visually (AV). All pairwise crossing of these factors produced six task conditions, entitled AAWord, AANonWord, AVWord, AVNonWord, VVWord, and VVNonWord. Lexical trial presentation and timing for each task are shown in Fig. 2(a–f). All tasks were generated using E-prime software (Psychology Software Tools, Pittsburgh, PA).

In general, participants first completed visual modality tasks, then cross-modality audio-visual tasks, and finally auditory modality tasks. When possible participants completed all tasks from one modality in a single day. Within a scan day, task and run order was counterbalanced across participants. Due to variation in task completion date, participant age is provided for every run in *participants.tsv* at the root level of the dataset. At session T2, participants completed VV tasks only.

Each task contained 96 word or non-word pairs categorized into four conditions: 24 pairs were orthographically similar and phonologically similar (O+ P+), 24 pairs were orthographically different and phonologically similar (O− P+), 24 pairs were orthographically similar and phonologically different (O+ P−), and 24 pairs were orthographically different and phonologically different (O− P−). All words were monosyllabic, have neither homophones nor homographs, and were matched across conditions for written word frequency in children^34^, the sum of their written bigram frequency, naming mean accuracy, and lexical decision mean accuracy^35^. The same word/non-word pairs were used across word and non-word tasks. Word/non-word pairs were presented sequentially each for 800 ms separated by a 200 ms inter-stimulus interval. Visually presented stimuli were presented in the center of the screen against a white background while auditorily presented stimuli were presented through sound attenuating headphones while a black fixation cross was presented in the center of the screen. After presentation of the second stimulus, participants were presented with a red fixation cross indicating that they should respond. Red fixation cross presentation time varied between 2200, 2600, and 3000 ms (400 ms jitter). Participants were able to respond as soon as the second stimulus was presented up until the beginning of the next trial.

**Fig. 3:**
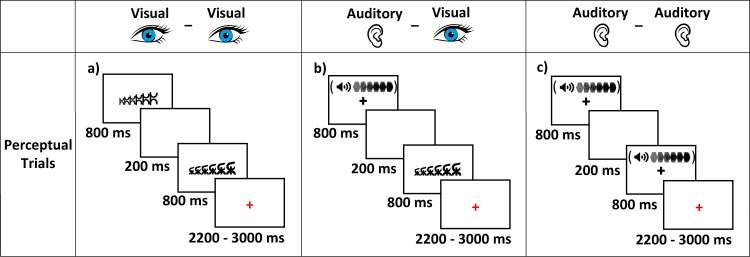
Perceptual trials stimuli and timing. Illustration of the stimuli and timing of perceptual trials used within tasks by sensory modality; (**a**) perceptual trial for visual-visual rhyming tasks, (**b**) perceptual trial for auditory-visual rhyming tasks, and (**c**) perceptual trial for auditory-auditory rhyming tasks.

In addition to lexical trials, each task contained 24 perceptual trials to control for sensory activation and 48 fixation trials to control for motor response only. In visual-visual perceptual trials, two sets of symbols were presented sequentially. Symbol sets were either increasing, decreasing, or steady in height from left to right. In these trials, participants were asked to judge if the two sets of symbols matched in height shape. In auditory-auditory perceptual trials, two tones were presented sequentially following the same timing as lexical trials. Tones were either increasing, decreasing, or steady in pitch. In these trials, participants were asked to judge if the two tones matched in pitch shape. In auditory-visual perceptual trials, participants were first presented with a tone and then with a set of symbols and were asked if the two stimuli matched in shape. Perceptual trial presentation and timing are shown in Fig. 3. Stimuli timing and response period for all perceptual trials was the same as lexical trials. In all tasks, fixation trials included two black crosses each presented for 800 ms separated by a 200 ms inter-stimulus interval followed by a blue fixation cross for 2200, 2600, or 3000 ms (400 ms jitter). Participants were instructed to press a button when they saw the blue cross. Each task contained 168 total trials that were divided into two 84 trial runs titled run-01 and run-02. Tasks were divided into two runs to reduce each functional scan time and maintain participant attention. Each run ended with the presentation of a black cross for 22000 ms. Trials were presented in a fixed pseudo-randomized order optimized by optseq 2 per each task^36^. Stimulus pair presentation order was counterbalanced across participants, with about half of participants seeing A_stim then B_stim and the other half seeing B_stim followed by A_stim.

All stimuli are provided in the stimuli folder at the root level of the dataset. Length of auditory non-word and word stimuli is provided in *NonWordAudDuration.tsv* and *WordAudDuration.tsv* respectively located within the stimuli folder. Table 7 provides information about the performance on each task at each session.

**Table 7:** Performance on fMRI tasks for T1 only and longitudinal MRI samples. Percentage accuracy and mean response time for correctly solved lexical trials for all tasks at each session.

		Session	T1 sample (n = 188)		Longitudinal MRI sample (n = 49)	
			Accuracy	Response Time	Accuracy	Response Time
Auditory-Auditory	Word	T1	70.1 (45.8)	1469 (520)	70.8 (45.5)	1466 (519)
	NonWord	T1	67.5 (46.8)	1445 (506)	68.2 (46.6)	1402 (553)
Auditory-Visual	Word	T1	65.5 (47.5)	1195 (498)	67.8 (46.7)	1216 (519)
	NonWord	T1	58.4 (49.3)	1234 (519)	59.4 (49.1)	1239 (528)
Visual-Visual	Word	T1	67.8 (46.7)	1326 (526)	73.4 (44.2)	1315 (508)
		T2	—	—	82.2 (38.3)	1113 (425)
	NonWord	T1	55.6 (49.7)	1322 (549)	58.8 (49.2)	1346 (537)
		T2	—	—	65.3 (47.6)	1155 (433)

## Data Records

This dataset is made public under the Creative Commons CCO license and hosted on the OpenNeuro platform (openneuro.org)^37^. The data is organized in accordance with the Brain Imaging Data Structure (BIDS) specification version 1.2.0^38^. BIDS is an organizational and naming convention for neuroimaging and behavioral data created to facilitate understanding and ease of use when sharing data. Openneuro.org provides a built-in BIDS validation tool that screens all uploaded datasets to ensure compliance with the BIDS specification. Any warnings generated by the BIDS validation tool for this dataset are explained in the known issues section of the README file included in the dataset.

All neuroimaging data is in the compressed Neuroimaging Informatics Technology Initiative (NIfTI) format (nii.gz), all tabular data files are in tab-separated values text file format (tsv), and all data dictionary descriptor files are in JavaScript object notation (json) format.

At the root level of the dataset, participant demographic information, including sex, and handedness, and age at standardized testing and each scan are provided in the *participants.tsv* file and these variables are further described in the accompanying data dictionary, *participants.json*. Psycho-educational assessment and questionnaire data as well as accompanying descriptive json files can be found in the *phenotype* folder, sorted by session and test. Neuroimaging data is located in individual subject folders labeled *sub-*<*ID*>, organized by session and imaging type. fMRI task behavioral event data is stored in the *func* folder in the appropriate *ses-*<*sessionID*> folder for each subject alongside their BOLD imaging data file for that same run. fMRI task behavioral data is compiled per trial and includes onset, duration, trial type, accuracy, response time, A stimulus, and B stimulus. Descriptions of parameters and event file column headers can be found at the root level of the dataset under *task-*<*task name*>*_bold.json*, and *task-*<*task name*>*_events.json* respectively. Online-only Table 1 provides a detailed description of the organization of all data records.

## Technical Validation

All psycho-educational tests were scored twice by trained research team members, and compared for reliability. In the case of discrepancy, a third scorer would review and conclude the correct score. Upon curation of the dataset all scores were reviewed to ensure no data entry errors had occurred. All identifying information in free response questions in the developmental history questionnaire were removed to protect confidentiality of participants.

Neuroimaging data were converted from standard DICOM to NifTI format using MRIConvert version 2.0. A documented bug in the MRICovert software stored repetition time inaccurately in the header of most files. Repetition time was corrected for all imaging modalities using the Analysis of Functional NeuroImages (AFNI) program nifti_tool^39^. Imaging parameters for structural and functional images were extracted from the DICOM headers and stored in a data dictionary json file at the root level of the dataset by imaging type and task.

Functional T2-weighted images were reoriented to the anterior commissure. All images were evaluated for movement due to high likelihood of in-scanner movement in pediatric populations. Scans that had greater than 25% of volumes reporting volume-to-volume motion of greater than 1.5 mm, as indicated by ArtRepair toolbox^40^, were removed from the dataset.

Facial features were scrubbed from all T1-weighted images by aligning the image to template space using FreeSurfer mri_robust_register, using an inverse registration on a template defacing mask, and then multiplying the transformed mask by the raw image^41^. Visual inspection confirmed that all facial features were completely removed and no part of the brain image was cut.

**Fig. 4:**
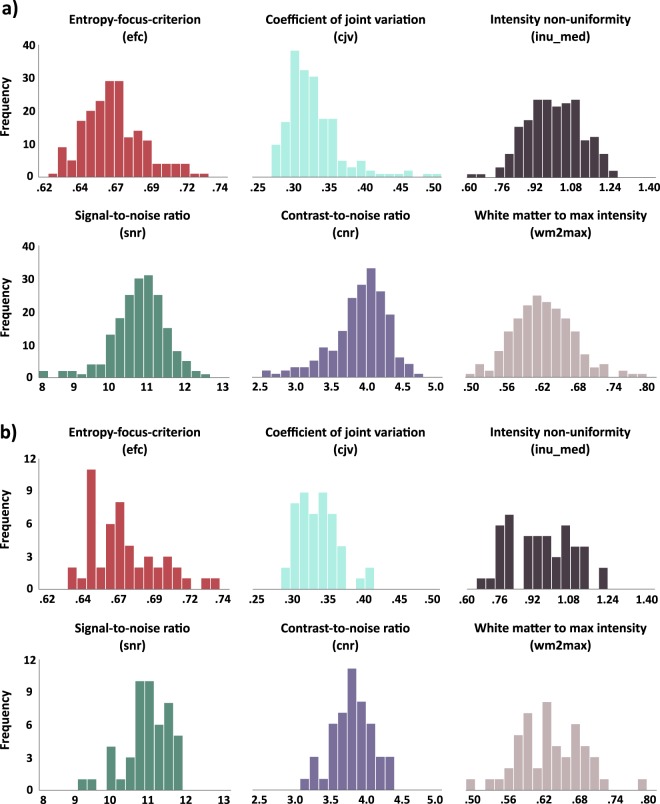
Distribution of quality metrics for T1-weighted MPRAGE data. Distribution of quality metrics for T1-weighted MPRAGE data (**a**) at session T1 (n = 188) and (**b**) session T2 (n = 49).

**Fig. 5:**
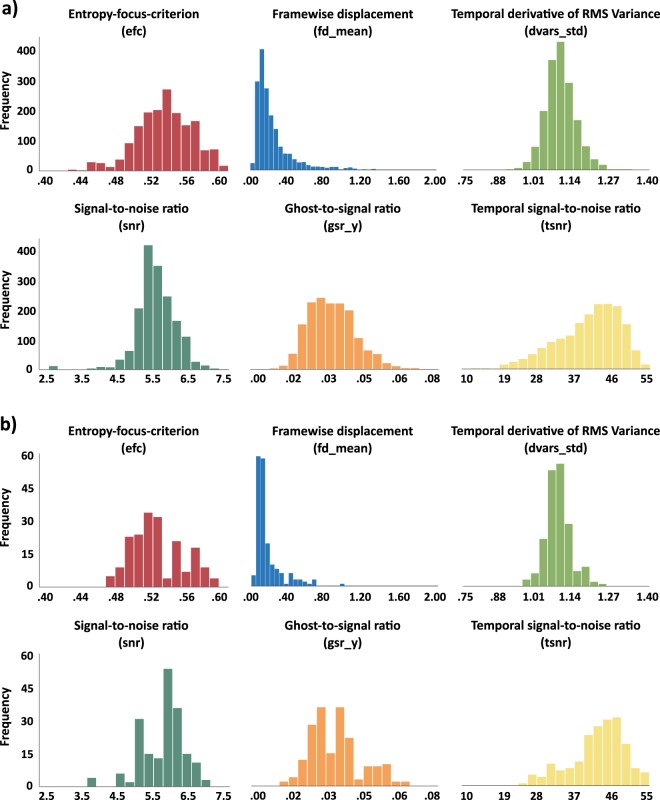
Distribution of quality metrics for T2-weighted fMRI data. Distribution of quality metrics for T2-weighted fMRI data (**a**) at session T1 (n = 1701) and (**b**) session T2 (n = 190).

**Fig. 6:**
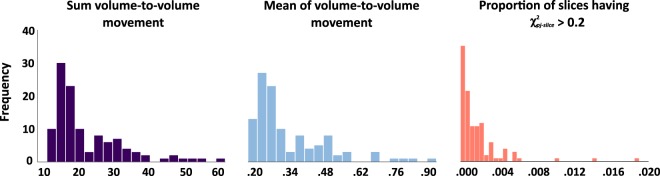
Distribution of quality metrics for diffusion weighted data. Distribution of quality metrics for diffusion weighted images collected at session T1 (n = 113).

After removal of facial features and high movement scans, all T1- and T2-weighted images were reviewed with the MRI Quality Control tool (MRIQC)^42^. MRIQC PDF reports of each image are included in the *derivatives/mriqc/reports* folder. Table 8 defines quality metrics displayed in Figs. 4–6. Figures 4 and 5 provide histogram representations of six quality control measures for T1- and T2-weighted images respectively. Image quality metrics were within ranges reported in previous datasets of similar age ranges, including Brain Correlates of Math Development^43,44^, and the Autistic Brain Imaging Data Exchange (ABIDE) dataset (https://mriqc.s3.amazonaws.com/abide/bold_group.html and https://mriqc.s3.amazonaws.com/abide/T1w_group.html).

**Table 8:** Description of selected quality control metrics. Descriptions of selected metrics from MRIQC and DTI QC pipeline output presented in figures X-X.

Image Type	Metric	Description
T1- and T2-weighted	Entropy-focus criterion (efc)	A measurement of ghosting and blurring caused by head motion. Lower values are better^46^.
	Signal-to-noise ratio (snr)	A measurement of quality of signal within the brain tissue. Higher values are better^47^.
T1-weighted	Coefficient of joint variation (cjv)	A measurement of noise indicating head motion and INU artifacts. Lower values are better^48^.
	Contrast-to-noise ratio (cnr)	A measurement of noise indicating separation of grey and white matter tissue distributions. Higher values are better^47^.
	Intensity non-uniformity median (inu_med)	A measurement of artifacts indicating the median of the bias field from INU correction. Values closer to 1.0 are better^49^.
	White-matter to maximum intensity ratio (wm2max)	A measurement of artifacts indicating the median intensity of white matter over the 95^th^ percentile of the total intensity distribution. Values between 0.6 and 0.8 are best^42^.
T2-weighted	Mean framewise displacement (fd_mean)	A measurement of movement indicating head movement across data acquisition calculated by realignment. Lower values are better^50^.
	Ghost-to-signal ratio (gsr_y)	A measurement of artifacts indicating the intensity of Nyquist ghost signal in the y-direction due to suboptimal EPI sequence calibrations. Lower values are better^51^.
	Normalized temporal derivative of RMS variance (dvars_std)	A measure of signal change across volumes indicating the normalized temporal derivative of variance across all voxels. Lower values are better^52^.
	Median temporal signal-to-noise ratio (tsnr)	A measurement of quality of signal calculated as median BOLD signal over temporal standard deviation. Higher values are better^42^.
Diffusion weighted	Volume-to-volume movement	“total movement” relative to previous volume using fsl eddy function eddy_movement_rms output^53^.
	${{\rm{\chi }}}_{pj-slice}^{2}$	Slicewise goodness of fit of image to diffusion model. Values above 0.2 are poor fitting and/or have high noise^45^.

In-scanner behavioral data were converted from raw E-prime data files to text files and then extracted for each subject and saved as tab separated values using python.

Quality of diffusion weighted images was assessed using a modified version of the pipeline proposed by Lauzon *et al*.^45^. PDF reports of each image are included in the *derivatives/dwi_QA* folder. Figure 6 shows mean and sum of volume-to-volume movement derived using FSL eddy RMS movement and proportion of slices having ${{\rm{\chi }}}_{pj-slice}^{2}$ greater than 0.2.

## Usage Notes

All data are publically available under Creative Commons CCO license. We encourage the use of this dataset for further analysis and publication under the requirement of citing this article and the dataset^37^. This dataset was successfully analyzed using SPM for fMRI and FSL for dwi analysis in previous publications^17–28^. We recommend that GLM analyses on these data incorporate chronological age or behavioral (e.g., reading age) measures as regressors of interest or non-interest to account for differences in time within-session and between-session as reported in Table 1. For those wishing to explore group contrasts, we recommend either finding matched sub-groups within these data, or computing residualized values for the fMRI data, after variance attributable to chronological age or other nuisance variables has been accounted for. Questions regarding this dataset can be directed to the corresponding author or posted as a comment on the OpenNeuro.org page for the dataset.

## Data Availability

Code used to create event data files from compiled E-prime data and to deface T1-weighted images are located in the code directory at the root level of the dataset. *reading-events-to-tsv.py* uses .csv containing merged data from all subjects per task and outputs events.tsv files into each subject folder as described in data records. *reading_deface.bash* and *multiply_by_mask.py* remove facial features from all T1-weighted images. *stims_checking.py* confirms that all stimuli referenced in participant events.tsv files exist in the stimuli directory at the root level of the dataset.

## Online-only Table

**Online-only Table 1:** Summary of data files location and names. Type, name, and description of all data files contained in dataset.

Data type	File name	Description
Demographics	participants.json	Description of column headers in participants.tsv
	participants.tsv	Subject demographics including age at standardized assessments, age at each scan, sex, and handedness
Standardized Assessments	phenotype/ses-<T#>/<test>.json	Description of standardized assessments and questionnaire variables
	phenotype/ses-<T#>/<test>.tsv	Standardized assessment and questionnaire data
Stimuli	stimuli/<word>.bmp	Lexical and perceptual visual stimuli
	stimuli/<word>.wav	Lexical and perceptual auditory stimuli
T1-weighted MPRAGE	T1w.json	MRI scanner parameters of anatomical scans
	sub-<ID>/ses-<T#>/anat/sub-<ID>_ses-<T#>_T1w.nii.gz	Anatomical scan at each session
Diffusion weighted images	dwi.json	MRI scanner parameters of diffusion weighted scans
	sub-<ID>/ses-T1/dwi/sub-<ID>_ses-T1_dwi.json	TR of subject specific diffusion weighted scan
	sub-<ID>/ses-T1/dwi/sub-<ID>_ses-T1_dwi.nii.gz	Diffusion weighted image
	sub-<ID>/ses-T1/dwi/sub-<ID>_ses-T1_dwi.bvec	Gradient vectors of subject specific diffusion weighted images
	sub-<ID>/ses-T1/dwi/sub-<ID>_ses-T1_dwi.bval	*b* values of subject specific diffusion weighted images
T2-weighted BOLD fMRI	task-<task name>_bold.json	Functional task description and MRI scanner parameters
	sub-<ID>/ses-<T#>/func/sub-<ID>_ses-<T#>_task-<task name>_run-<num>_bold.nii.gz	Functional scan for each run of each task
fMRI behavioral task	task-<task name>_events.json	Description of column headers for fMRI behavioral data in events.tsv files
	sub-<ID>/ses-<T#>/func/sub-<ID>_ses-<T#>_task-<task name>_events.tsv	fMRI behavioral data including trial onset, type, accuracy, response time, and stimuli per trial of each run for each task
Quality Control	derivatives/dwi_qc	PDF output of quality assessment of dwi data
	derivatives/mriqc	Derivatives.html, reports.html, and overall quality metrics.csv files from mriqc software output
Code	code/multiply_by_mask.py	Function used by reading_deface.bash to deface anatomical data
	code/reading-events-to-tsv.py	Code used to convert fMRI behavioral e-prime exported text files to *events.tsv files for each subject
	code/reading_deface.bash	Code used to deface anatomical data
Dataset Description	dataset_description.json	Description of dataset including authors, BIDS version, publication citations, funding, and license
	README	Explanation of known issues with data and warnings from BIDS validation
	CHANGES	Dataset changes by version

Note: <T#> is session identifier (T1 or T2), <test> is abbreviation of standardized assessment or questionnaire (adhd-rs, ctopp, dev_hist_questionnaire, towre, wasi, wj-iii), <word> is stimuli name as provided in behavioral task data file (i.e. *bride.wav*), <ID> corresponds to subject numbers (001-188), <task name> is fmri task name as described in methods (AAWord, AANonWord, AVWord, AVNonWord, VVWord, or VVNonWord), <num> is run number for the given fMRI task (01 or 02).
